# Screen Time Patterns and Their Impact on Academic Performance: A Prospective Study Among Phase 1 MBBS Students

**DOI:** 10.7759/cureus.101440

**Published:** 2026-01-13

**Authors:** Hannah Lukram, Reeta Baishya, Sankalpa Chakma

**Affiliations:** 1 Physiology, Gauhati Medical College and Hospital, Guwahati, IND

**Keywords:** academic performance/grades, digital learning, digital screen time, medical school students, northeast india, screen time

## Abstract

Background

The increasing use of smartphones and digital devices among medical students has raised concerns about their impact on academic performance. While academic screen time may support learning, excessive recreational usage can have a negative effect. As Phase 1 MBBS students bear a heavy academic load, they are an ideal group in which to study this balance.

Methods

A prospective observational study was conducted among 102 Phase 1 MBBS students, in which screen time was manually collected from the students’ device records. The duration of the study was four consecutive weeks in January 2025. The screen times were then categorised into academic and non-academic use. The academic performance of the students was measured based on their first-trimester marks in February 2025. To complement this, participants also reported their preferred mode of study, whether physical books, digital books (PDFs), online video content, or a mixed approach. Furthermore, the applications or platforms used for study and entertainment were noted and verified. Statistical analysis was performed using Spearman’s correlation and Kruskal-Wallis tests. Ethical approval and informed consent were obtained prior to the study.

Results

Among 102 students, the mean daily screen time was 5.9 ± 2.0 hours, which could be categorised into 2.9 ± 1.2 hours for academic use and 3.0 ± 1.6 hours for non-academic use. Academic screen time correlated positively with examination marks (ρ = 0.541, p < 0.001), while non-academic use correlated negatively (ρ = -0.575, p < 0.001). However, total screen time showed no significant correlation (ρ = -0.091, p = 0.364). Students preferring digital books/PDFs scored the highest mean (58.3%), while those relying mainly on physical books scored the lowest (45.1%, p = 0.037). As far as the relationship between the different applications used for study or entertainment and marks is concerned, no significant differences in marks were observed (p = 0.311 and p = 0.511, respectively).

Conclusion

The impact of screen time on learning depends more on its purpose than on its duration. While academic use was linked with better exam performance, recreational use was associated with poorer outcomes. Hence, promoting focused academic use of digital devices, while limiting distractions, may improve learning outcomes in medical students.

## Introduction

Medical education, especially during the first year, is highly demanding. Students struggle to maintain their cognitive focus, time management, and overall well-being. During both study and leisure time, students end up using a variety of digital devices extensively. In fact, in 2023, the global average daily digital screen time was estimated to be 6 hours and 37 minutes, with that for younger people often exceeding eight hours per day [1].

Despite these observations, the association between screen time and academic performance remains inconsistent across studies. Some report a negative link, while others find no significant relationship, and a few note potential benefits under specific conditions. Hence, screen time usage is quite nuanced and must be distinguished between academic and non-academic usage [2-4].

For medical students, this distinction is particularly relevant. On one hand, devices facilitate access to lectures, digital textbooks, online videos, and academic apps, offering flexibility and efficiency in learning. On the other hand, non-academic activities, such as social media, gaming, and entertainment apps, may compete with study time and thus reduce productivity [2,5]. Understanding how these different patterns of use affect academic outcomes is especially important in the formative Phase 1 MBBS stage, when students are adapting to a demanding curriculum and establishing long-term learning habits [3,6].

Objectives of the study

The primary objective of this study is to assess the association between academic and non-academic screen time and academic performance. The secondary objective of this study is to analyse the effect of gender, preferred mode of study, and use of different applications in relation to the association between academic and non-academic screen time and academic performance.

## Materials and methods

Study site 

The study was conducted at Gauhati Medical College and Hospital, a government institute and one of the oldest medical colleges in Northeast India. Located in Guwahati, the capital city of Assam, the college, at the time of the study, admitted 200 MBBS students annually from all parts of the country. The study was conducted from January to February 2025.

Study participants

The study included Phase 1 MBBS students aged 18 or older who provided consent and owned a smartphone or tablet capable of tracking screen time. Students who declined participation or had incomplete weekly screen time data were excluded from the analysis.

Sample size

The sample size was calculated to detect a correlation between screen time patterns and academic marks using the formula:

$N = \left[ \frac{(Z_{\alpha} + Z_{\beta})}{C} \right]^2 + 3$ Where $C = 0.5 \times \ln \left( \frac{1 + r}{1 - r} \right)$, substituting the values for a correlation coefficient of r = 0.3, the minimum required sample size was calculated to be 85. To enhance reliability and account for potential data loss, a total of 102 Phase 1 MBBS students were included in the study.

Study procedure 

The study was approved by the Institutional Ethics Committee of Gauhati Medical College and Hospital on December 26, 2024 (MC No.: 190/2007/PtII/Dec.2024/8). Written informed consent was obtained from all participants prior to data collection. No identifiable information was recorded or stored.

Data collection 

Participants were contacted after academic hours, and written informed consent was obtained prior to data collection. Weekly screen time data were collected manually from each participant’s smartphone or tablet using in-built features, such as “Screen Time” (for iOS devices) and “Digital Wellbeing” (for Android devices). The data were recorded over a four-week period in January. If a participant used more than one smartphone or tablet, screen time from all devices was included.

Screen time was then categorised into academic screen time and non-academic screen time. Academic screen time was the time spent on academic applications, e-books, PDFs, online video lectures, and other study-related resources. Non-academic screen time was the time spent on social media, gaming, entertainment, and other non-academic activities.

Academic performance was assessed using the marks obtained in the first-trimester examinations (Anatomy, Physiology, and Biochemistry), conducted in February. These data were collected manually from the respective academic departments.

No online forms or self-reported surveys (such as Google Forms) were used. All data were obtained directly from devices and departmental records to ensure accuracy and reliability.

Statistical analysis 

The categorical data distributions have been described using frequency distribution tables with percentage frequencies, while the continuous data have been summarised using the arithmetic mean and standard deviation. Spearman’s correlation analysis has been carried out to study the association between screen time and average marks, while comparison of average marks with respect to the various independent variables was done using the Kruskal-Wallis test. All tests have been conducted at a 0.05 significance level. The statistics have been computed using IBM SPSS Statistics for Windows, Version 20 (Released 2011; IBM Corp., Armonk, NY, USA).

## Results

Out of 102 Phase 1 MBBS students, 72 (70.6%) were male and 30 (29.4%) were female. Table 1 shows the descriptive statistics for screen time and academic performance. Average daily screen time was 5.9 hours, with nearly equal distribution between academic (2.9 hours) and non-academic (3.0 hours) use. Females spent more time on academic activities and obtained higher average marks (56.7%) compared to males (49.6%). 

**Table 1:** Distribution of screen time and marks among the participants

	Overall		Female		Male	
	Mean	SD	Mean	SD	Mean	SD
Total screen time (hours)	5.9	2.0	5.7	2.0	6.0	2.0
Academic screen time (hours)	2.9	1.2	3.3	1.3	2.7	1.1
Non-academic screen time (hours)	3.0	1.6	2.4	1.0	3.2	1.7
Average marks scored	51.7	12.6	56.7	9.5	49.6	13.2

Table 2 shows the correlation between total, academic, and non-academic screen time and average marks among all 102 participants. Total screen time had no significant association with performance (ρ = -0.091, p = 0.364). In contrast, academic screen time was positively correlated with marks (ρ = 0.541, p < 0.001), whereas non-academic screen time showed a significant negative correlation (ρ = -0.575, p < 0.001). This suggests that the type of screen use, rather than overall duration, is more important in determining academic outcomes.

**Table 2:** Correlation between screen time and marks

	Spearman’s rho (ρ)	p-value
Total screen time vis-à-vis average marks scored	-0.091	0.364
Academic screen time vis-à-vis average marks scored	0.541	<0.001 (significant)
Non-academic screen time vis-à-vis average marks scored	-0.575	<0.001 (significant)

Table 3 presents subgroup analysis by gender. Among females, academic screen time showed a positive but non-significant correlation with marks (ρ = 0.333, p = 0.072), while non-academic screen time had a significant negative correlation (ρ = -0.409, p = 0.025). Among males, both trends were stronger: academic screen time correlated positively with marks (ρ = 0.598, p < 0.001), and non-academic screen time correlated negatively (ρ = -0.578, p < 0.001). However, due to the low sample size, this gender-based analysis of the correlation between screen time and academic performance cannot be clearly established. 

**Table 3:** Correlation of screen time with academic performance stratified by gender

	Total screen time vis-à-vis average marks scored		Academic screen time vis-à-vis average marks scored		Non-academic screen time vis-à-vis average marks scored	
	Spearman’s rho (ρ)	p-value	Spearman’s rho (ρ)	p-value	Spearman’s rho (ρ)	p-value
Female	0.004	0.983	0.333	0.072	-0.409	0.025 (significant)
Male	-0.138	0.248	0.598	<0.001 (significant)	-0.578	<0.001 (significant)

Table 4 shows the preferred study mode of the participants and their mean marks. Most participants preferred mixed study modes (68.6%), indicating that they combined multiple resources, such as physical books, digital books/PDFs, and online videos, for their preparation. Participants who primarily used digital books/PDFs had the highest mean marks (58.3%), while those relying solely on physical books had the lowest mean marks (45.1%). The reported test statistic (8.439), with p = 0.037, indicates a statistically significant difference in mean marks across study-mode groups (Kruskal-Wallis test).

**Table 4:** Preferred study mode and mean marks

Study mode	N	Mean	Std. deviation	Minimum	Maximum	Test statistic	p-value
Digital books/PDF	5 (4.9%)	58.3	8.7	49.8	70	8.439	0.037 (significant)
Online video content	8 (7.8%)	53.3	8.5	36.3	63.2		
Physical books	19 (18.6%)	45.1	9.6	26.0	60.7		
Mixed modes	70 (68.6%)	52.8	13.4	18.7	73.8		
Total	102 (100%)	51.7	12.6	18.7	73.8		

Table 5 represents the study applications used and the mean marks. YouTube is the most common study platform (69.6%), reflecting its role as an educational video resource, followed by Med Ed (12.7%). The test statistic (4.778, p = 0.311) shows no significant difference in marks across different study apps, indicating that the choice of app did not meaningfully impact academic performance. However, descriptively, Telegram users had lower mean marks (45.3%).

**Table 5:** Study applications used and mean marks

Study app used	N	Mean	Std. deviation	Minimum	Maximum	Test statistic	p-value
ChatGPT	4 (3.9%)	54.6	14.0	34.8	67.3	4.778	0.311 (no significant)
Med Ed	13 (12.7%)	53.6	12.4	31.7	70.7		
Telegram	12 (11.8%)	45.3	11.4	30.3	65.3		
YouTube	71 (69.6%)	52.4	12.7	18.7	73.8		
Others	2 (2%)	45.7	13.2	36.3	55.0		
Total	102 (100%)	51.7	12.6	18.7	73.8		

Table 6 represents the applications used for entertainment and the mean marks. Among entertainment applications, Instagram was the most frequently used (49%), followed by YouTube (30.4%). Although the mean examination scores appeared higher among WhatsApp users (56%), statistical testing showed no significant differences in academic performance between application groups (test statistic = 5.262, p = 0.511). Thus, the choice of entertainment applications did not appear to influence examination outcomes.

**Table 6:** Applications used for entertainment and mean marks

App used for entertainment	N	Mean	Std. deviation	Minimum	Maximum	Test statistic	p-value
Games	4 (3.9%)	50.8	22.1	18.7	69.0	5.262	0.511 (no significant)
Instagram	50 (49%)	50.0	12.8	21.3	73.8		
OTT platforms	2 (2%)	46.0	14.1	36.0	56.0		
WhatsApp	11 (10.8%)	56.0	12.7	30.3	70.0		
YouTube	31 (30.4%)	53.0	11.8	26.0	70.7		
Others	4 (3.9%)	53.5	5.6	48.5	60.7		
Total	102 (100%)	51.7	12.6	18.7	73.8		

## Discussion

In this study of Phase 1 MBBS students, screen time emerged as a double-edged factor influencing academic performance. Academic screen time showed a clear positive correlation with examination scores. However, non-academic screen time was strongly and negatively correlated with average marks scored. Interestingly, total screen time was not related to performance, suggesting that the purpose of device use matters more than the duration. Further, neither the choice of study apps nor the type of entertainment apps showed any significant effect on marks, though descriptive trends were observed. Gender analysis revealed that the benefit of academic screen time was stronger among males, while the negative effect of non-academic use was consistent across both genders. Most students reported relying on a mixed study mode, combining digital and traditional resources, a pattern that was also observed among MBBS students in the Delhi/NCR region [7]. 

These findings are consistent with a growing body of research emphasising that not all screen time is equal. Several international studies have shown that excessive non-academic screen use, such as social media, gaming, and entertainment, is associated with poorer academic outcomes and reduced attention span [2,4,8]. A large study in China, for example, reported that higher time spent on gaming and internet surfing correlated with lower standardised test scores across subjects [2]. A meta-analysis also confirmed a small but significant negative association between excessive smartphone use and grade point average (GPA) [8]. Similarly, recent evidence among university students notes that prolonged recreational digital screen use can impair learning efficiency [4]. Specifically, among undergraduate nursing students in Pakistan, a significant negative correlation was observed between daily screen time and GPA and exam scores [9]. Also, a study conducted among children in India demonstrated a clear link not only between excess screen time and poorer academic performance, but also anxiety [10]. Thus, our finding that non-academic screen time strongly predicted poorer performance is well aligned with global evidence.

On the other hand, the positive role of academic screen use deserves emphasis. Devices, when used for accessing lectures, digital books, and study apps, appear to provide a meaningful advantage. Previous research often failed to distinguish academic from non-academic use, which may explain why many studies reported mixed or inconsistent results. For instance, an Indian study from JSS Medical College found that bedtime gadget use, rather than total daily use, was associated with poorer academic scores, mediated by sleep disruption [6]. However, excessive academic screen time may lead to burnout in medical students [11]. Therefore, the quality, timing, and amount of device use are critical. Academic screen time represents active, directed learning, while non-academic use is more passive and distracting.

The gender difference we observed in our study - that academic screen time is more beneficial among males - may not be statistically significant due to the small sample size; however, it is worth noting that another study, conducted on 2,582 students in grades 8-11, also found the same [12]. This may be due to differences in how male and female students engage with technology. 

As such, this is one of the few studies in the country to objectively separate academic and non-academic screen time and correlate each with examination performance in medical students. Conducted at Gauhati Medical College and Hospital, one of the premier institutes with one of the largest MBBS intakes in India, the study underscores the importance of the purpose of screen use in determining academic outcomes.

Limitations 

Being conducted at a single institution among Phase 1 MBBS students, the findings may not be generalisable to students from other medical colleges or different years of training. The study also assessed screen time over a four-week period and academic performance based on a single trimester examination, which may not capture long-term patterns or cumulative academic outcomes. Although device-based features such as “Screen Time” and “Digital Wellbeing” were used to improve accuracy, certain data, particularly from laptops and televisions, were relied upon partly on participant recall, introducing potential bias. In addition, confounding factors such as baseline academic performance, socioeconomic status, mental health status, and sleep were not factored in during the study. Therefore, future studies with a larger sample size and longer follow-up can address the limitations of this study. 

## Conclusions

In summary, our study shows that, in early medical education, purposeful academic use of screens can be beneficial, while recreational use carries an academic cost. Differentiating between types of screen time is therefore crucial. Encouraging students to channel digital device use towards learning, while limiting entertainment use, may improve performance and support healthier academic habits.
